# Progress in Surgery

**Published:** 1897-04-10

**Authors:** 


**Radiography in Surgery.**—The practical application of the Roentgen rays has been greatly developed during the past few months. The successful reduction in the time of exposure to 4½ seconds has been accomplished by A. W. Goodspeed,[17] by improvements in the arrangement of the exciting apparatus, in the form of Crooke's tubes employed, and in the development of a specially sensitized photographic plate. The beautiful skiagraphs produced in *International Medical Magazine*, for June, 1896, of the head and upper and lower parts of the trunk are commented upon by W. W. Keen,[18] as being (at the date) very remarkable. The aids of the new process for elucidating points of surgica diagnosis have been the subject of a series of articles by W. Turner[19] and a brief *résumé* up to date (October, 1896) is given by Sydney Rowland.[20] The latter says as to its uses in the extremities that these are the regions in which so far the process has proved itself of most value. *A priori*, it might be seen that the limbs would prove themselves most adaptable to skiagraphic purposes, for in them there is the greatest proportion of opaque bony tissue to transparent flesh, and in them fractures and dislocations, for the elucidation of which the process is particularly useful, are most frequent. Taking the joints and bones of the extremities in order, he has had cases of rheumatoid arthritis and displaced epiphysis, in both of which great assistance was afforded by the use of the X rays. But it is in the elbow-joint that the greatest use will probably be found for the skiagraph in surgery. Owing to the complexity of its structure, this joint offers more conditions of difficult diagnosis than perhaps any other in the body. Fractures, separated epiphyses, epicondylar fractures, and many other intricate combinations of lesion which occur in or around this joint, can all be revealed with the greatest clearness. The joint is one very easily skiagraphed, even in the most muscular subject; owing to its mesial position in the extremity, any view or series of views can be obtained. He, therefore, like many other surgeons, confidently expects that, as regards this region of the body at least, the systematic use of the X rays will leave no more cases to suffer from the evil effects of erroneous diagnosis. Two cases of a halfpenny in the œsophagus, revealed by the same process, are recorded and figured by F. Johnston and C. T. Holland,[21] while a third by N. Raw[22] shows the appearance before successful removal. The latter author has also given[23] an excellent skiagram of the normal anatomy of the trunk and upper extremities of a boy aged six, in which foreign bodies had been artifically introduced. Another case of bullet embedded in the tissues of the neck is represented by R. G. Le Conte,[24] and demonstrates clearly how precisely bullets may be cut for which cannot be located by any other means.

**Diseases of Bones and Joints.** — In an article on traumatic hæmarthrosis of the knee-joint, John O'Conor[25] expresses the opinion that effusion of blood following injuries to the knee-joint is much more common than is ordinarily supposed. He traverses Mr. Barker's operation for recent fractures of the patella so far as it applies to the removal of the effused blood. With a narrow-bladed knife Barker makes two punctures, one above and the other below the patella, he then "squeezes" and "kneads" the joint, and by such manipulation professes to "effectually" empty it. O'Conor says that to anyone accustomed to evacuate blood from joints by free incision, this procedure must appear an uncertain, if not an impossible task, for there are some parts of the joint where squeezing and kneading cannot possibly dislodge the coagulating blood. He recommends that in the majority of cases an aspirating needle should be introduced in order to arrive at a correct diagnosis—an incision should immediately be made into the synovial pouch, and every particle of blood and clot removed; in case of fracture into the joint the fragments should be examined, and, if necessary, reduced and fixed. A gauze drain should be inserted for a few days in order to make certain that no further reaccumulation shall take place. The external wound is afterwards closed by "waiting" sutures. When union is sufficiently firm active movement and massage should be commenced. Boisseau de Rocher[26] has treated tuberculous suppuration and chronic osteomyelitis by electrolysis of a solution of sodium bromide within the tissues. The abscess cavity or the bone cleared of sequestra is filled with the solution by means of a platinum trocar which forms the anode of a constant current, the kathode being a large moist pad applied in the neighbourhood. The current decomposes the solution—bromine, hydrobromic acid, and oxygen compounds being formed. The author reports several very successful cases. During the last few years surgeons have inclined more and more towards conservative measures in the treatment of tuberculosis of the joints; operations with many are becoming the exception rather than the rule. Freeman[27] expresses his views on the treatment by injection of iodoform, and describes the method of sterilising the iodoform and mode of injecting the joint cavity by means of an ordinary hypodermic syringe; while Campanini[28] gives details of several examples of surgical tuberculosis and tuberculous joint disease successfully treated by Durante's method, viz., by injecting iodine. Ziématzky[29] advocates Lannelongue's method of injecting a solution of chloride of zinc. It gives, he says, excellent results when the tuberculous disease is attacked at an early stage, and before the development of suppuration and extensive caries. Lannelongue considers that this sets up a zone of firm connective tissue, which shuts off the tuberculous deposit from the rest of the organism; but Ziématzky explains the good results by the chloride of zinc acting directly upon and killing the tubercle bacilli. That muscle atrophy is a constant and prominent symptom in tuberculous joint disease is a well-known fact, but no explanation of this atrophy has been suggested hitherto, except a vague and mysterious reflex influence. However, A. G. Miller[30] has come to the conclusion that the contraction of the arteries, with the consequent diminished blood supply, which have been seen and proved to exist, is the cause of the muscle atrophy. Cathcart[31] inclines to the usual explanation that the atrophy is due to non-use of the limb. From an examination of the blood in forty-three cases of tuberculosis of the bones and joints, J. Dane[32] finds that in most cases the number of red corpuscles is not decreased, but the percentage of hæmoglobin is affected, giving rise to a mild degree of chlorosis. The leucocyte count seems to bear no relation to the temperature. High counts, especially in hip disease, point to the probability that there is, or shortly will be, an abscess formation, but low counts do not preclude the presence of abscess, especially in cases of long standing. An interesting example of necrosis of the os calsis in a boy aged seven and a-half years is recorded by E. Owen[33]. There was septic osteomyelitis with necrosis of the diaphyses of the tibiæ and left ulna. After removal of the sequestra the case did well.

[17] International Medical Mag., June, 1896, p. 317. [18] Ibid., p. 319. [19] Lancet, June 20, 1896 (et seq.) [20] Brit. Med. Jour., Oct. 3, 1896, p. 925. [21] Brit. Med. Jour., Dec. 5, 1896, p. 1,677. [22] Ibid., p. 1,678. [23] Lancet, Nov. 21, 1896, p. 1,416. [24] Annals of Surgery, Aug., 1896, p. 217. [25] Glasgow Med. Jour., Dec., 1896; and Annals of Surgery, Nov. 1896, p. 621. [26] Epit. B. Med. Journ., Nov. 21, 1896; and Archiv. Gén. de Méd., Sept. 1896. [27] Therapeutic Gazette, Sept. 15, 1896, p. 616. [28] Epit. B. M. J., Dec. 5, 1896; and Il Policlinico, Oct. 1, 1896. [29] Epit. B. M. Jour., Oct. 3, 1893; and Rev. de Chir., Aug., 1896. [30] Edinburgh Med. Journ., Sept., 1896, p. 220. [31] Ibid., Oct., 1896, p. 357. [32] Amer. Medico-Surgical Bulletin, Sept. 12, 1896; and Boston Med. and Surg. Jour., cxxxiv., No. 24. [33] Lancet, Jan. 2, 1897, p. 37.

